# Meals based on vegetable protein sources (beans and peas) are more satiating than meals based on animal protein sources (veal and pork) – a randomized cross-over meal test study

**DOI:** 10.3402/fnr.v60.32634

**Published:** 2016-10-19

**Authors:** Marlene D. Kristensen, Nathalie T. Bendsen, Sheena M. Christensen, Arne Astrup, Anne Raben

**Affiliations:** 1 Department of Nutrition, Exercise and Sports, Faculty of Science, University of Copenhagen, Copenhagen, Denmark; 2 Novo Nordisk A/S, Bagsværd, Denmark; 3 Ferring Farmaceuticals A/S, Copenhagen, Denmark

**Keywords:** legumes, appetite, fullness, hunger, dietary fiber, ad libitum

## Abstract

**Background:**

Recent nutrition recommendations advocate a reduction in protein from animal sources (pork, beef) because of environmental concerns. Instead, protein from vegetable sources (beans, peas) should be increased. However, little is known about the effect of these vegetable protein sources on appetite regulation.

**Objective:**

To examine whether meals based on vegetable protein sources (beans/peas) are comparable to meals based on animal protein sources (veal/pork) regarding meal-induced appetite sensations.

**Design:**

In total, 43 healthy, normal-weight, young men completed this randomized, double-blind, placebo-controlled, three-way, cross-over meal test. The meals (all 3.5 MJ, 28 energy-% (E%) fat) were either high protein based on veal and pork meat, HP-Meat (19 E% protein, 53 E% carbohydrate, 6 g fiber/100 g); high protein based on legumes (beans and peas), HP-Legume (19 E% protein, 53 E% carbohydrate, 25 g fiber/100 g); or low-protein based on legumes, LP-Legume (9 E% protein, 62 E% carbohydrate, 10 g fiber/100 g). Subjective appetite sensations were recorded at baseline and every half hour using visual analog scales until the *ad libitum* meal 3 h after the test meal. Repeated measurements analyses and summary analyses were performed using ANCOVA (SAS).

**Results:**

HP-Legume induced lower composite appetite score, hunger, prospective food consumption, and higher fullness compared to HP-Meat and LP-Legume (*p*<0.05). Furthermore, satiety was higher after HP-Legume than HP-Meat (*p*<0.05). When adjusting for palatability, HP-Legume still resulted in lower composite appetite scores, hunger, prospective consumption, and higher fullness compared to HP-Meat (*p*<0.05). Furthermore, HP-Legume induced higher fullness than LP-Legume (*p*<0.05). A 12% and 13% lower energy intake, respectively, was seen after HP-Legume compared to HP-Meat or LP-Legume (*p*<0.01).

**Conclusion:**

Vegetable-based meals (beans/peas) influenced appetite sensations favorably compared to animal-based meals (pork/veal) with similar energy and protein content, but lower fiber content. Interestingly, a vegetable-based meal with low protein content was as satiating and palatable as an animal-based meal with high protein content.

Randomized controlled trials and some prospective cohort studies have demonstrated that a high protein intake can induce increased weight loss and improve weight maintenance after weight loss compared to a low protein intake (1–5). This effect appears to be associated with reduced feelings of hunger and increased satiety (6, 7), although increased energy expenditure may also be a contributing factor (8, 9).

In recent years, increasing attention has been put on the environmental impact of different foods, and it has become clear that meat products may contribute with more negative effects, for example, production of greenhouse gasses and CO_2_ emission, than vegetable products (10–12). Therefore, a replacement of protein from animal sources such as beef and pork with protein from vegetable sources, such as legumes, would be an environmental-friendly approach. Such an approach would also lead to an increased intake of dietary fiber.

Recently, the Nordic Nutrition Recommendations were revised to reflect the issue on environmental impact of food production. Thus, a reduction in consumption of protein from animal sources such as beef and pork and an increase in vegetable sources such as legumes and pulses was recommended (13). Furthermore, focus should be on whole foods rather than single macronutrients.

When comparing CO_2_ emission for legumes with meat, the CO_2_ equivalent (in kg) is increased with a factor of 30 (12). Despite this, the consumption of protein-rich legumes is very low among Danes compared to the intake of, for example, red meat. Adults in Denmark consume on average 137 g of red meat and meat products per day (14), whereas the intake of legumes is approximately 7 g per day (15). The obstacles that could be accountable for the low intake could be palatability, gastrointestinal discomfort, but probably even more importantly, that legumes are not part of a typical Western dietary culture. A study investigated how chick pea supplementation in an Australian diet affected satiation and bowel health (16). They found that perceived satiation and perceived bowel function were improved. However, inconvenience and gastrointestinal upset were considered to discourage legume consumption.

Very little is still known about the effect of Nordic-grown vegetable sources of protein on appetite and body weight regulation. The aim of the present study was therefore to examine whether a meal based on vegetable sources (legumes: beans and peas) was comparable to a meal based on natural animal sources (pork and veal) regarding acute meal-induced appetite sensations and *ad libitum* energy intake.

## Methods and materials

### Study design

The study was designed as a randomized, double-blind, placebo-controlled, three-way cross-over intervention. Each meal test was separated by a washout period of at least 2 weeks. Subjects were instructed to refrain from alcohol consumption and intense physical activity for the 24 h proceeding each test day. In the evening before the test day, the subjects consumed a 4.0 MJ standardized evening meal (50.3 energy-% (E%) carbohydrate, 33 E% fat, 16.7 E% protein), consisting of pork and vegetable stew with rice (prepared by kitchen staff at the department) before 8:00 p.m. After this time subjects fasted (consumption of half a liter of water was allowed).

On each test day, the subjects met fasting in the morning at the department. After voiding, they were weighed to the nearest 0.05 kg on a decimal scale (Lindeltronic 8000, Copenhagen, Denmark). Before the test meal was served, subjects filled in the first visual analog scales (VASs). The test meal was served and consumed within 15 min. After the meal, subjects filled in VAS at time points 15, 30, 60, 90, 120, 150, and 180 min. VAS for palatability of the test meals were filled in immediately after finishing the meal. Three hours after the test meal, an *ad libitum* lunch meal was served, and subjects were instructed to eat until they felt comfortably satiated. The *ad libitum* lunch consisted of Pasta Bolognese and was served with 300 ml water. The meal had an energy content of 961 kJ/100 g and a macronutrient composition of 55 E% carbohydrates, 30 E% fat, and 15 E% protein. Food intake was registered and energy intake calculated. The subjects were not allowed to consume any other foods or drinks throughout the test day, but were allowed to read, listen to the radio, or use their computer.

### Subjects

Healthy young men were recruited through university intranet systems and advertisements on websites. Subjects should be normal-weight to moderately overweight (body mass index (BMI) 22–28 kg/m^2^), between 18 and 40 years of age, and free of any chronic health conditions. Subjects who smoked, were athletes (>10 h exercise/week), used regular medication, had food allergies, or used dietary supplements were excluded. In total, 60 subjects were screened; 7 subjects did not meet the inclusion/exclusion criteria, and 5 withdrew consent before starting the study. Thus, 48 subjects started the study. The subjects were given both verbal and written information, whereupon all gave written consent. Subjects meeting all the inclusion criteria were randomly allocated to the three meals. Meal sequences were generated by hand to make an even number of each sequence and a computer was used to generate a list of these sequences in random order. The list was kept by the investigator and not disclosed to the study coordinator who enrolled the subjects in the study.

The study was carried out at the Department of Nutrition, Exercise and Sports, Faculty of Science, University of Copenhagen, Frederiksberg, Denmark, April–July 2011. It was approved by the Municipal Ethical Committee of The Capital Region of Denmark to be in accordance with the Helsinki-II declaration. Subjects received ~300 US$ as compensation on completion of all the test days. The trial was registered at clinicaltrials.gov as NCT01345487.

### Test meals

The test meals were given as breakfast meals and each provided 3.5 MJ (Table 1). Three test meals were compared: one with a high protein content from meat, HP-Meat (19 E% protein, 53 E% carbohydrate, 28 E% fat, 6 g fiber/100 g); one with a high protein content from legumes, HP-Legume (19 E% protein, 53 E% carbohydrate, 28 E% fat, 25 g fiber/100 g); and one with a low protein content from legumes, LP-Legume (9 E% protein, 62 E% carbohydrate, 28 E% fat, 10 g fiber/100 g). The meals were all composed of oven-baked patties, made of veal and pork meat (HP-Meat) or of fava beans (HP-Legume and LP-Legume), and a mash of either potato (HP-Meat), split peas (HP-Legume), or a combination of the two (LP-Legume) (Table 1). Water was added to the recipes of the HP-Legume and LP-Legume meals in order to achieve similar serving weight and energy density of all three meals. The final serving weight was 591 g for the three meals. The test meals were served with 300 ml of drinking water. The meals were prepared by the experienced kitchen staff at the department, but were blinded when served by the study responsible to the subjects. The energy content and nutrient composition of the test meals were calculated using Dankost 3000 dietary assessment software (Danish Catering Center, Herlev, Denmark). The software is based on the food database from the Danish Technical University. The energy factors used are 17 kJ/g for protein, 37 kJ/g for fat, 17 kJ/g for available carbohydrate, and 8 kJ/g for dietary fiber. Fiber content was analyzed by a standard method (17) and protein content was measured using the Kjeldahl method (18).

**Table 1:** Test meal ingredients and nutrient content (raw weight)

HP-Meat	g	HP-Legumes	g	LP-Legumes	g
Veal and pork patties		Fava bean patties		Fava bean patties	
Veal/pork, minced, 4% fat	135	Fava beans, dried	100	Fava beans, dried	29
Potato, raw, shredded	84			Potato, raw, shredded	124
Potato flour	6			Potato flour	39
Rapeseed oil	7	Rapeseed oil	10	Rapeseed oil	10
		Butter	5	Butter	4
Onions, fresh, diced	10	Onions, fresh, diced	10	Onions, fresh, diced	10
Breadcrumbs	20	Flour	5	Flour	8
Garlic, parsley, salt, cumin		Garlic, parsley, salt, cumin		Garlic, parsley, salt, cumin	
Mashed potatoes		Mash of split peas		Mash of split peas	
Potato	254	Split peas, dried	90	Split peas, dried	32
Potato flour	36			Potato	176
Butter	13	Butter	12	Butter	14
Rapeseed oil	2				
Salt		Salt and vinegar		Salt and vinegar	
Tomato ketchup	20	Tomato ketchup	20	Tomato ketchup	20
					
3,546 kJ		3,552 kJ		3,545 kJ	
19 E% protein		19 E% protein		9 E% protein	
39 g protein/100 g^a^		38 g protein/100 g^a^		18 g protein/100 g^a^	
28 E% fat		28 E% fat		28 E% fat	
53 E% carbohydrate		53 E% carbohydrate		62 E% carbohydrate	
6 g fiber/100 g^b^		25 g fiber/100 g^b^		10 g fiber/100 g^b^	
Serving weight: 591 g		Serving weight: 591 g		Serving weight: 591 g	

Water was added to the HP-Legume and LP-Legume recipes to achieve similar serving weight of all three meals.

a Measured values. A 90% digestibility of protein from the vegetable sources was used in the calculations

b Analyzed values. Analyzed by a standard method (12). E%: Energy%. HP-Legume: high protein (19 E%) from legumes and HP-Meat: high protein (19 E%) from veal and pork meat. LP-Legume: low protein (9 E%) from legumes.

### Measurements of subjective appetite

VASs were used to assess subjective perception of palatability of the test meals as well as appetite sensations before and for 180 min after consumption of the test meals. VAS, 100 mm in length with words anchored at each end, expressing the most positive and the most negative rating, were used to answer questions regarding the meal (palatability, taste, aroma, physical appearance, and off- taste) and appetite sensations (satiety, hunger, fullness, prospective food consumption, thirst, well-being, and desire to eat something fatty, sweet, salty, or meaty). The four standardized questions in relation to satiety feelings were: How satisfied do you feel? (‘completely empty’ to ‘cannot eat another bite’); How hungry do you feel? (‘not hungry at all’ to ‘as hungry as I have ever felt’); How full do you feel? (‘not full at all’ to ‘totally full’); and How much do you think you can eat? (‘nothing at all’ to ‘a large amount’). The four questions related to desire were: Do you feel like eating something sweet? (‘yes, very much’ to ‘no, not at all’); Do you feel like eating something salty? (‘yes, very much’ to ‘no, not at all’); Do you feel like eating something fatty? (‘yes, very much’ to ‘no, not at all’); Do you feel like eating something meaty/fishy? (‘yes, very much’ to ‘no, not at all’). The questionnaires were made as small booklets showing only one question at a time. Subjects were not allowed to discuss or compare their ratings with each other and could not refer to their previous ratings when filling in the VAS booklets. The use, reproducibility, and validity of the VAS have been described before by Flint et al. (19).

In order to integrate the different feelings of appetite, a composite appetite score was calculated for each time point. The score has been described before (20) and is calculated with this formula:

[Satiety+hunger+(100-fullness)+(100-prospective food consumption)]/4.

### Sample size calculation

Power calculation was done based on the primary end-point, composite appetite score (area under the curve [AUC]) as well as on *ad libitum* energy intake. From unpublished data we estimated that a minimum of 42 subjects was required to detect an absolute difference of 275 mm*60 min in composite appetite score and 385 kJ in *ad libitum* energy intake between diets with a statistical power of 80%, and a two-sided significance level of 5%. To allow for an estimated ~10% drop out rate, 48 participants were recruited.

### Statistical analyses

The statistical analyses were performed on participants who completed the intervention (*n*=43) using SAS 9.3 (SAS Institute, Cary, NC, USA). The statistical significance level was defined as *p*<0.05. Data are presented as estimated means±SEM, unless otherwise stated. The appetite scores were analyzed using repeated measurements and AUC, calculated by the trapezoidal rule. Repeated measurements analyses were done using ANCOVA-type linear mixed models including a time-treatment interaction and adjusting for age, BMI, order of treatments, baseline appetite score, and with and without adjustment for assessed overall palatability of test meal. Additionally, differences between subjects were accounted for by means of random effects. AUCs were analyzed using ANCOVA models including treatment and adjusting for age, BMI, order of treatments, baseline appetite score, and with and without adjustment for assessed overall palatability of test meal. *Ad libitum* energy intake was analyzed using an ANCOVA model including treatment and adjusting for age, BMI, and order of treatments. For the repeated measurements we saw no significant time×treatment interactions. If a significant overall treatment effect was found, *post hoc* tests with Tukey-Kramer adjustment for multiplicity were carried out to identify significant differences between meals.

## Results

Of the 48 included subjects, 43 completed all three meal sessions and were included in the analyses (Age: 24.4±4.8 years; height: 182.6±7.2 cm; body weight: 76.8±7.3 kg; BMI: 23.0±2.1 kg/m^2^). Two subjects withdrew after the first test meal because of gastrointestinal discomfort and three withdrew after the second test meal because of lack of time.

### Ratings of palatability of test meals

The test meals were assessed in terms of physical appearance, aroma, taste, off-taste, and overall palatability (Table 2). The palatability of the HP-Legume meal was rated significantly poorer than that of the other two meals (*p*<0.0001). No difference between the HP-Meat and the LP-Legume meal was observed for any of these parameters. The palatability of the *ad libitum* meal was rated similarly after the three test meals (data not shown).

**Table 2:** Palatability assessments and AUCs of thirst and well-being of the three test meals (mm)

	HP-Meat	HP-Legume	LP-Legume
Palatability of test meal, mm	54±3^a^	29±3^b^	55±4^a^
Taste of test meal, mm	60±3^a^	31±3^b^	57±4^a^
Aroma of test meal, mm	61±3^a^	43±3^b^	57±3^a^
Physical appearance of test meal, mm	45±4^a^	29±3^b^	46±3^a^
Off-taste of test meal, mm	27±4	37±4	30±4
Thirst AUC, mm×min	9,923±470^ab^	10,892±424^b^	9,968±518^a^
Well-being AUC, mm×min	11,325±313^a^	10,528±331^b^	11,106±328^a^

Numbers with different letters are significantly different (*p*<0.05). AUC: Area under the curve. HP-Meat: high protein (19 E%) from veal and pork meat (*n*=43). HP-Legume: high protein (19 E%) from legumes. LP-Legume: low protein (9 E%) from legumes.

### Ratings of subjective appetite sensations

**Fig. 1:**
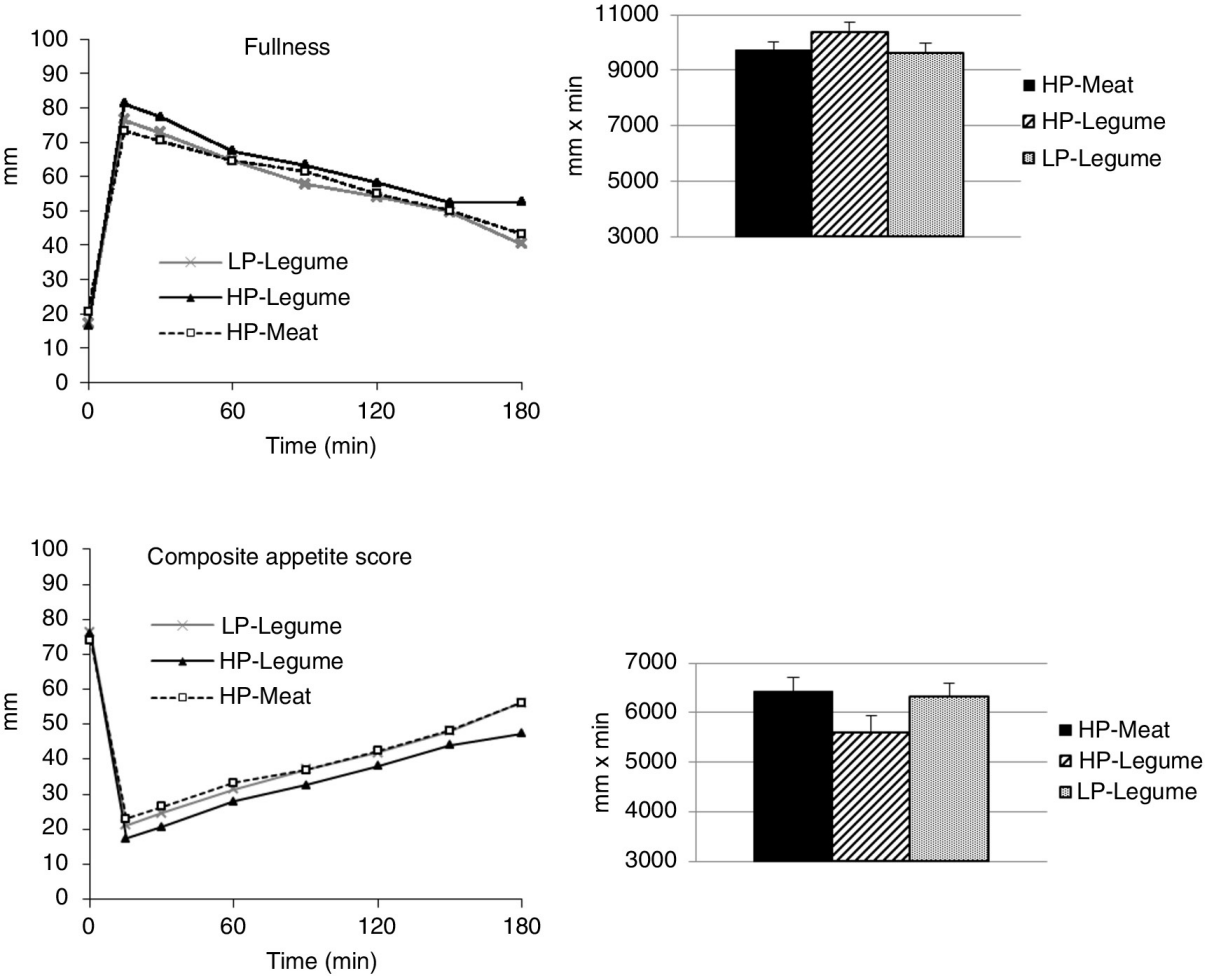
Left panel: Line chart of unadjusted mean ratings. Right panel: Bar chart of mean (±SEM) area under the curves (AUC) for fullness and composite appetite score during 3 h after 3 iso-caloric test meals (*n*=43). *Fullness*: Repeated measures (with and without adjustment for palatability): Meal *p*<0.05, Time *p*<0.0001. Fullness higher after HP-Legume compared with HP-Meat and LP-Legume (*p*<0.05). AUC: HP-Legumes>LP–Legumes (*p*<0.05). No differences in AUC after adjusting for palatability. *Composite appetite score* (CAS): Repeated measures (with and without adjustment for palatability): Meal *p*<0.05, Time *p*<0.0001. HP-Legume lower CAS than HP-Meat and LP-Legume, *p*<0.05. After adjustment for palatability: HP-Legume lower than HP-Meat, *p*<0.05 (but not than LP-Legume). AUC: HP-Legumes lower than LP-Legumes, *p*<0.05. No differences in AUC after adjustment for palatability. HP-Meat: high protein (19 E%) from veal and pork meat. HP-Legume: high protein (19 E%) from legumes. LP-Legume: low protein (9 E%) from legumes.

Postprandial responses in appetite ratings are illustrated by the scores for composite appetite and fullness sensations (Fig. 1). A significant effect of time was seen for all appetite parameters (*p*<0.0001). Furthermore, an effect of meal was seen for all appetite parameters (*p*<0.05). After adjusting for overall palatability, an effect of meal was still seen for composite appetite score (*p*<0.01), prospective food consumption (*p*<0.05), hunger (*p*<0.01), and fullness (*p*<0.05).

Pairwise comparisons (without adjusting for overall palatability) showed that the HP-Legume meal resulted in lower ratings for composite appetite scores, hunger, and prospective food intake compared to the HP-Meat and LP-Legume meal (*p<*0.05 for all). Furthermore, the HP-Legume meal was significantly more satiating than the HP-Meat meal (*p<*0.05), but not than the LP-Legume meal (*p*>0.05). When adjusting for overall palatability of the test meals, the HP-Legume meal still resulted in lower ratings of composite appetite, hunger, and prospective food intake compared to the HP-Meat meal (*p<*0.05), but not the LP-Legume meal. Fullness after the HP-Legume meal was significantly higher than after the HP-Meat and LP-Legume meal, both with and without adjustment for overall palatability (*p<*0.05 for both).

The AUCs showed lower composite appetite score, hunger, and prospective food intake and higher satiety after the HP-Legume meal compared to the HP-Meat meal (*p<*0.05 for all). Additionally, the HP-Legume meal caused lower composite appetite fullness ratings compared to the LP-Legume meal (*p<*0.05 for both). When adjusting for overall palatability of the test meal no significant differences in AUCs were seen any more.

### Ad libitum energy intake

**Fig. 2:**
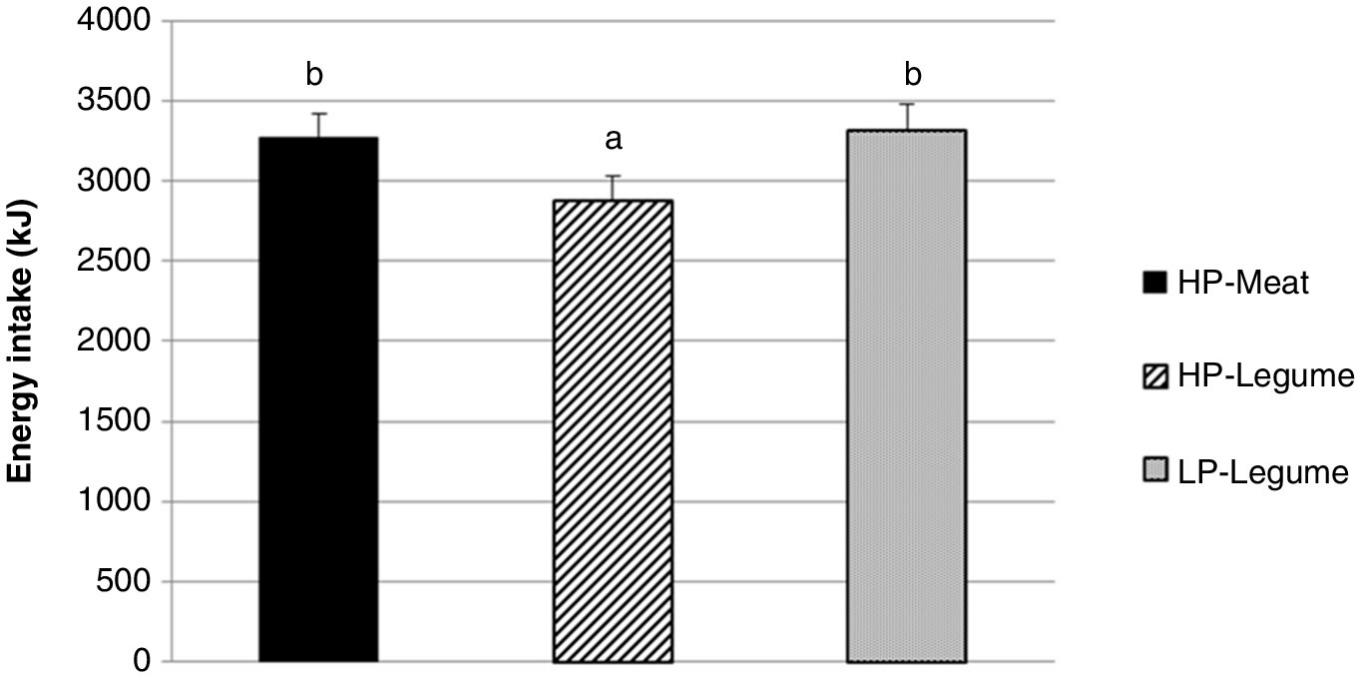
*Ad libitum* energy intake (mean±SEM) 3 h after the test meals. *Ad libitum* energy intake was reduced by 12% and 13%, respectively, after consumption of the HP-Legume meal, compared to HP-Meat and LP-Legume (*p*<0.01 for both). HP-Meat: high protein (19 E%) from veal and pork meat. HP-Legume: high protein (19 E%) from legumes. LP-Legume: low protein (9 E%) from legumes.

A significant meal effect was seen for *ad libitum* energy intake 3 h after the test meal (*p<*0.001). Thus, energy intake was reduced by 400 kJ and 440 kJ (12 and 13%), (*p<*0.001) after the HP-Legume meal compared to the HP-Meat and LP-Legume meals, respectively (Fig. 2).

### Ratings of subjective sensory-specific desires

The overall well-being of the subjects was rated lower after consumption of the HP-Legume meal compared with the two other meals (*p<*0.01 for pairwise comparisons) and the HP-Legume meal induced greater feelings of thirst than the LP-Legume meal (*p<*0.05) (Table 2). The specific desires to eat something fatty, sweet, salty, or meaty were not different between meals (*p*>0.05, data not shown).

## Discussion

The current study showed that a high-protein meal based on vegetable sources (legumes: beans and peas), induced higher post-meal satiety ratings and lower energy intake compared with a high-protein meal based on animal sources (veal and pork). Even more interestingly, a low-protein meal based on legumes was both as satiating and as palatable as a high-protein meal based on veal and pork.

Although the vegetable meal was more favorable with regard to appetite regulation, this does not document that vegetable protein *per se* is more satiating than animal protein. Because of the choice of natural food sources, the HP vegetable meal had a higher fiber content than the HP animal meal, and this may have induced a higher satiety feeling. Also palatability of the HP legume meal was rated lower than the other two meals and this partly affected the results. Still, if choosing such vegetable protein sources, our results indicate that it is possible to obtain similar satiety, but for less protein, than with animal sources such as those chosen here.

To our knowledge, this is the first study to compare the effect on appetite regulation of vegetable meals based on beans and peas with animal meals based on pork and veal. There are several previous studies in this field. However, they have mainly examined soy vs dairy (whey, casein, milk) and used supplements and not real foods as test meals (e.g. soy or whey dissolved in water) (21). A few studies have compared pea vs dairy, but also as supplements (22, 23). Only a handful of studies have used real foods (solid) foods or meals and can therefore more readily be compared with the current study (24–28).

Douglas et al. (24) evaluated the effect of meals with beef or soy protein (34 E%) on appetite and hormonal signals in 21 young, normal-weight adults. Meals were macronutrient and fiber-matched or serving-size-matched. There were no significant differences in postprandial hunger, fullness, peptide YY (PYY), glucagon-like peptide-1 responses, or *ad libitum* energy intake after the beef or soy lunches, whether matched for macronutrients and fiber or for serving size. Another short-term cross-over study investigated iso-energetic, high-protein breakfasts and lunches (30 E% protein) based on meat (lean beef and ham) or soy (soy protein powder mixed in a shake) in 12 normal- or overweight men and women (25). No differences were seen in satiety during an 8 h postprandial period, however, lack of power could perhaps explain the lack of differences here (19).

Similar results were found in a more long-term cross-over study, which compared vegetarian (soy protein or soy-textured vegetable protein) and meat-based (chicken and beef) high-protein diets (30 E% protein) (23). Twenty overweight or obese men were included and each diet period lasted for 14 days with three meals per day. No differences in daily rated subjective appetite sensations or motivation to eat were found between the two diets (26). Still, differences in rated pleasantness of the diets might have biased the results.

Bayham et al. (27) compared egg vs cereal in energy- and macronutrient-matched breakfast meals (20 E% protein) using a 7-day cross-over design and 21 overweight men and women. They found that fullness and PYY was higher after egg vs cereal on the first study day, but there were no differences after 7 days’ exposure.

The last meal test study using foods compared protein (17 E%) from mycoprotein (plant), tofu (plant), or chicken (meat) served as a pasta dish 4 h after breakfast to 42 overweight women in a cross-over design (28). The meals were isocaloric and macronutrient-matched, but the mycoprotein meal contained twice as much fiber (6 g) as the other two meals (3 g). Subjective appetite sensations did not differ, but *ad libitum* energy intake was significantly higher after intake of the chicken meal vs either of the two plant meals. This finding partly corresponds to our present findings.

As mentioned above, soy is in general the most studied vegetable protein source, whereas no or few studies have looked at brown beans or green peas, more commonly eaten in the Nordic countries. Furthermore, soy was most often given as a protein powder mixture. It can be assumed that the different vegetable sources matter when comparing studies, as the amino acid composition is not identical. Thus, in contrast to other vegetable protein sources, soy contains all essential amino acids, although in relatively small amounts. One previous study comparing soy with pea (supplements) did not see any differences, though (29). Because only 12 subjects were included here, lack of power could, however, also explain this finding (19).

One prospective cohort study which investigated the intake of animal and plant protein and subsequent weight changes is worth mentioning. The result was based on 89,432 European men and women who filled in country-specific food frequency questionnaires and were followed for a mean of 6.5 years. A positive association between animal protein and subsequent weight gain was found, but this was not the case with vegetable protein (30). This indicates less satiety from eating animal protein than plant protein. However, it is not possible to exclude whether other macronutrients or dietary factors in the food items (i.e. dietary fiber) influenced the results (30, 31).

A recent meta-analysis supports that dietary pulses can be a beneficial weight-loss strategy (32). Thus, analyses of data from 21 studies showed an overall significant weight reduction of −0.34 kg when diets included dietary pulses compared with when diets did not. The analyses also suggested that dietary pulses may reduce body fat mass.

The relatively large difference in fiber content between our test meals most likely plays an important role with regard to the satiating effects (33, 34). The fact that there was no difference between the LP-Legume meal and the HP-Meat meal indicates that a low-protein meal with fiber-rich beans/peas is as satiating as a high-protein meal with pork/veal and suggests that vegetable protein sources can be efficient alternatives for weight regulation with less use of protein than with animal protein sources. Thus, in respect to satiety, a high content of dietary fibers may be as effective as a high content of protein, when using real foods.

The strengths and weaknesses of the present study should be considered. We consider it a strength that we used real foods in realistic meals. Thus, such meals could be expected to be consumed outside the clinical setting. This is in contrast to many of the previous studies, where more unnatural meals and supplements were used. It could be argued, though, that a weakness in our design was that dietary fiber content was then not matched in the two HP meals. However, this is a consequence of using real foods. If a 2×2 factorial design had been used, we might have been able to obtain a clearer picture of the factor of interest (protein) without changing other factors that could influence the primary outcome.

The current study also highlights that a high protein level is difficult to obtain solely from vegetable sources without compromising palatability. Statistical analyses were therefore done with and without adjustment for palatability of the test meals. This resulted in similar, although less significant results for the appetite parameters. Such a finding is in line with the notion that food not considered palatable is perceived as more satiating than palatable food (35). It may be that our study subjects were not familiar with vegetable-based foods and meals and that this influenced their palatability ratings. Energy density and weight or volume are also factors important for energy intake and appetite sensations. We strived to compose meals with similar weight and hence energy density. Therefore, these factors were not likely to be of importance in the present study.

## Conclusion

In conclusion, a vegetable-based meal (beans/peas) influenced appetite sensations and energy intake favorably compared to an animal-based meal (pork/veal) with similar energy and protein content. Interestingly, a vegetable-based meal with low protein content was as satiating and palatable as an animal-based meal with high protein content. Differences in dietary fiber and palatability are likely important contributing factors.
